# Is sperm FISH analysis still useful for Robertsonian translocations? Meiotic analysis for 23 patients and review of the literature

**DOI:** 10.1186/s12610-018-0069-z

**Published:** 2018-05-07

**Authors:** Anna Lamotte, Guillaume Martinez, Françoise Devillard, Jean-Pascal Hograindleur, Véronique Satre, Charles Coutton, Radu Harbuz, Florence Amblard, James Lespinasse, Mehdi Benchaib, Julien Bessonnat, Sophie Brouillet, Sylviane Hennebicq

**Affiliations:** 1 CHU de Grenoble, UF de Biologie de la procréation, F-38000 Grenoble, France; 2 CHU de Grenoble, UF de Génétique Chromosomique, F-38000 Grenoble, France; 3 Université Grenoble Alpes, F-38000 Grenoble, France; 4 Team ‘Genetics Epigenetics and Therapies of Infertility’, Institute for Advanced Biosciences INSERM U1209, CNRS UMR5309, F-38000 Grenoble, France; 5 Service de génétique CH de Chambéry, Chambery, F-38000 France; 6 Centre d’AMP, HFME, CHU de Lyon, Lyon, F-69000 France

**Keywords:** Robertsonian translocation, Sperm FISH, Meiotic segregation, Spermatozoa, Preimplantation genetic diagnosis, Translocation robertsonienne, hybridation in situ, ségrégation méiotique, spermatozoïde, diagnostic génétique préimplantatoire

## Abstract

**Background:**

Robertsonian translocations (RobT) are common structural chromosome rearrangements where carriers display a majority of chromosomally balanced spermatozoa from alternate segregation mode. According to some monotony observed in the rates of balanced segregation, is sperm FISH analysis obsolete for RobT carriers?

**Methods:**

Retrospective cohort research study on 23 patients analyzed in our center from 2003 to 2017 and compared to the data of 187 patients in literature from 1983 to 2017.

Robertsonian translocation carriers were divided in six groups according to the chromosomes involved in the translocation: 9 patients from our center and 107 from literature carrying 45,XY,der(13;14) karyotype, 3 and 35 patients respectively with 45,XY,der(14;21), 5 and 11 patients respectively with 45,XY,der(13;15), 4 and 7 patients respectively with 45,XY,der(14;15), 1 and 4 patients respectively with 45,XY,der(13;22),and 1 and 10 patients respectively with 45,XY,der(14;22).

**Results:**

Alternate segregation mode is predominant in our group of Robertsonian translocation carriers with 73.45% ±8.05 of balanced spermatozoa (min 50.92%; max 89.99%). These results are compliant with the data from literature for all translocations types (*p* > 0.05) and are consistent among the different types of Robertsonian translocations (*p* > 0.05) except for der(13;15) that exhibit lower balanced spermatozoa rates (*p* < 0.05 versus der(13;14), der(14;21), (13;21) and der(15;22)). Normozoospermic patients also display a significantly (*p* < 0.01) higher rate of balanced sperm cells than patients with abnormal seminograms whatever the defect implied.

**Conclusions:**

According to the discrepancies observed between der(13;15) and all the other Rob T carriers, the differences observed among patients presenting normal and abnormal sperm parameters and the input in genetical counselling, sperm FISH does not seem obsolete for these patients. Moreover, it seems important to collect more data for rare RobT.

## Résumé

**Contexte:**

Le mode de ségrégation chromosomique le plus fréquemment observé chez les patients porteurs de translocation robertsonienne est. un mode équilibré. Les données semblent varier peu selon la translocation analysée. La relative constance des résultats dans le cas de ces translocations robertsoniennes rend elle inutile ces analyses chromosomiques pour ces patients?

**Patients et méthodes:**

Nous avons analysé de façon rétrospective les données spermatiques et de ségrégation méiotique de 23 patients porteurs de translocation robertsonienne, de 2003 à 2017 et comparé les résultats observés à ceux décrits dans la littérature pour 187 patients.

**Résultats:**

Le mode de ségrégation alterne est. prépondérant dans notre série de patients avec 73.45% ±8.05 de spermatozoïdes équilibrés (min 50.92%; max 89.99%). Ces résultats sont en accord avec les données de la littérature, toutes translocations confondues et selon le type de translocation (*p* > 0.05) sauf pour la translocation der(13;15) où ces taux sont significativement plus faibles (*p* < 0.05 vs der(13;14), der(14;21), (13;21) et der(15;22)). Nous observons également des taux de spermatozoïdes équilibrés significativement plus élevés chez les patients à spermogramme normal (*p* < 0.01).

**Conclusions:**

Les différences observées dans les taux d’aneuploïdies entre les translocations der(13;15) et les autres translocations robertsoniennes et entre les porteurs de translocation à spermogramme normal ou altéré, et l’utilité de ces données dans le conseil génétique conduisent à poursuivre l’analyse systématique de la ségrégation méiotique pour les patients porteurs de translocations robertsoniennes et ceci particulièrement pour les translocations rares.

## Background

Robertsonian translocation (RobT) is a frequent structural chromosomal aberration with an incidence of 1.23 per thousand births [1]. Carriers present a karyotype with 45 chromosomes resulting from centromeric fusion of two acrocentric chromosomes (13; 14; 15; 21 or 22). Most common Robertsonian translocations are der (13;14) and der(14;21) with a frequency of 73% and 10% respectively [2]. Unbalanced segregation of these chromosomes through meiosis can result in recurrent pregnancy loss if the unbalanced chromosomal content is not viable, or birth of a child with severe malformations and mental retardation in case of viability. The prevalence of RobT carriers in recurrent pregnancy loss and infertile male population are at least ten times higher (respectively 1.1% and 3% versus 0,1%) than in general population [2–5]. Knowing the rates of balanced and unbalanced segregation including the different types of unbalanced modes is thus of great importance in genetic counselling for these couples. Moreover, male carriers can present oligoasthenoteratozoospermia leading to procreation issues.

**Fig. 1:**
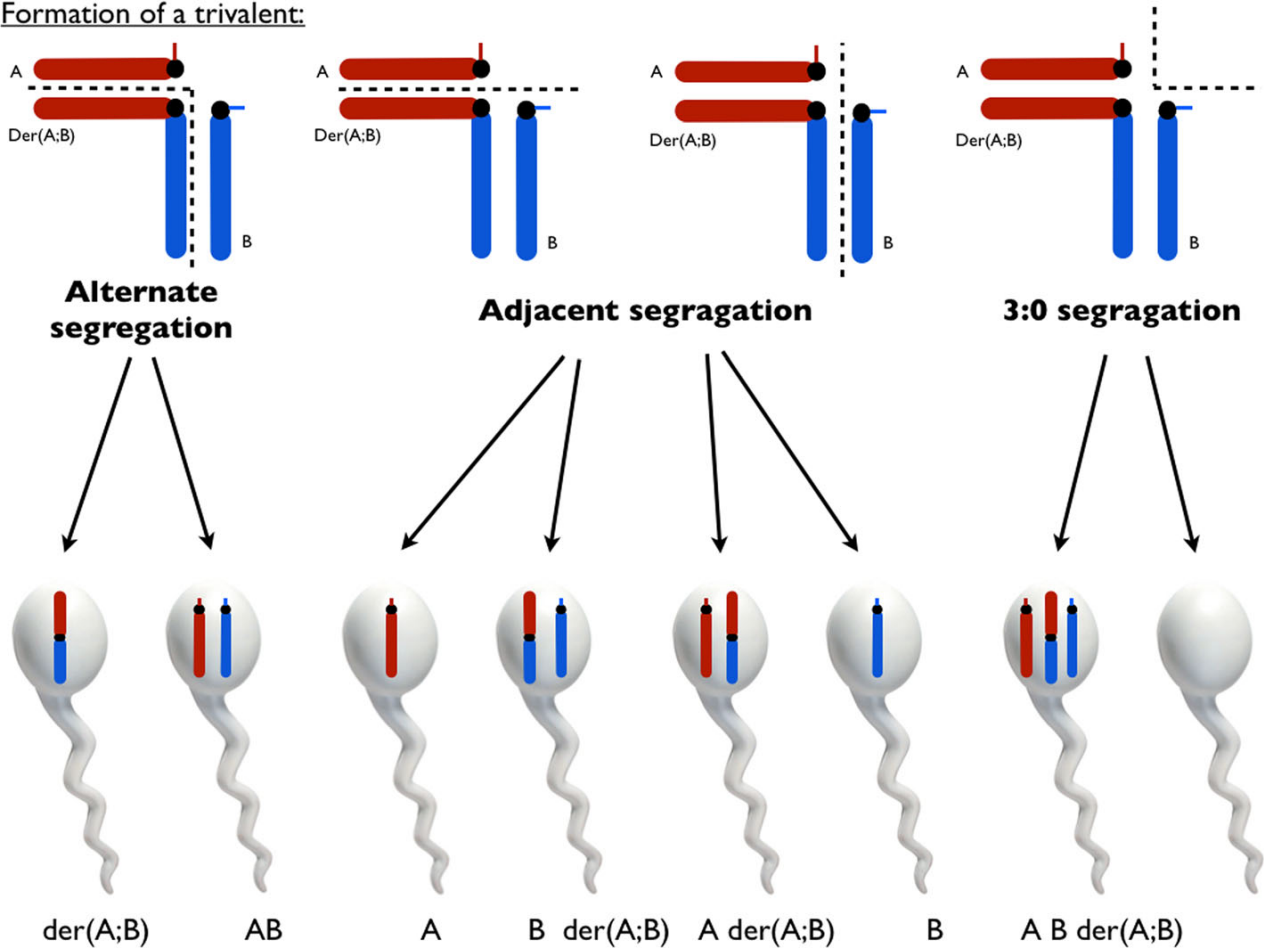
Formation of trivalent and its segregation in meiosis

During meiosis, pairing and segregation is possible through formation of a trivalent during prophase I (Fig. 1). Alternate segregation results in two balanced gametes containing either normal chromosomes A and B or the derivate der(A;B). FISH analysis does not allow differentiating these, neither in sperm, nor in the embryos. The karyotype of the conceptus is then either normal or presents the same translocation as the parent, possibly leading to abnormality in the child’s offspring at adulthood. The adjacent segregation modes lead either to sperm nullosomy or sperm disomy. In case of nullosomy, the conceptus presents a monosomy which is not viable, while in case of a sperm disomy, the conceptus presents a trisomy, which can be viable (from several hours to several years or more in trisomy 21). The 3:0 mode of segregation leads to sperm double nullosomy or disomy, leading to unviable monosomic or trisomic conceptus. Detailed analysis of the sperm chromosomal content can thus help genetical counselling through a quantification of (i) the chances of a viable pregnancy (balanced content of sperm and conceptus) and the risk of (ii) recurrent pregnancy loss (unviable monosomy or trisomy) or (iii) possibly viable trisomy. The risk of unbalanced conceptus highlights the importance of chromosomal meiotic segregation analysis. Preimplantation genetic diagnosis (PGD) for RobT carriers reduces the risk of pregnancy loss or multiple congenital anomalies and intellectual disability (MCA-ID) through selection and transfer of normal/balanced embryos.

The first analyses of meiotic segregation variants were done by heterospecific oocyte fertilization followed by sperm karyotyping. This technique was long and fastidious. It only allowed the analysis of a few number of sperm, moreover restricted to the fertile ones. One advantage of this technique was to distinguish between normal and balanced sperm. Development of fluorescence in-situ hybridization (FISH) technique has simplified the analysis of sperm chromosomal content and has enabled to collect numerous data on meiotic segregation and balanced and unbalanced rearrangements. This technique combined to automated slides scanning allows the analysis of a large number of sperm cells and is for several years used in routine practice.

The primary objective of this study was to assess the variability of meiotic segregation in sperm of RobT carriers. We thus, analyzed 23 new carriers and literature data of 187 patients. We also looked for factors influencing meiotic segregation rates.

## Methods

### Patients

Twenty three male patients aged 26 to 40 years, carrying a RobT, were included in this retrospective cohort study. They consulted for fertility issues in the genetic and procreation department of university hospital of Grenoble between january 2003 and april 2017, except for three of them who were referred by three other french centers (service de génétique, CHU de Reims; service de génétique, CH de Chambéry; centre d’AMP, HFME, CHU de Lyon, France).

Karyotype performed on blood cells was 45,XY,der(13;14)(q10;q10) in 9 patients, 45,XY,der(13;15)(q10;q10) in 5 patients, 45,XY,der(14;15)(q10;q10) in 4 patients, 45,XY,der(14;21)(q10;q10) in 3 patients, 45,XY,der(13;22)(q10;q10) in one patient and 45,XY,der(14;22)(q10;q10) in one patient.

Sperm FISH analyses performed between 2004 and 2006, as a research project, were submitted to a signed inform consent of all the patients with approval of the study by the ethic committee of the University Hospital of Grenoble. Since 2006, the analysis was achieved as a routine test, ruled by a signed informed genetic consent for all patients. The sperm preparation and sperm FISH techniques remained identical over the entire period of study.

### Sperm preparation

Semen samples were collected in a sterile container after masturbation. Liquefaction was obtained after 30 min at 37 °C. Sperm concentration, motility and morphology were determined according to WHO criteria (World Health Organization, 1999 for the analyses done until 2009 and WHO, 2010 for the analyses performed later on) [6].

### Sperm FISH technique

Samples were washed twice with 5 ml of phosphate-buffered saline (PBS) 1X and fixed in a methanol/acetic acid (3:1, *v*/v) solution. Cells were spread on Superfrost© (Kindler, Freidburg Germany) slides and air dried at room temperature. Sperm head decondensation was performed in NaOH 1 M solution, followed by two washes in 2X standard saline citrate (SSC) and dehydration in a 70, 90% and pure ethanol solution. Samples were then hybridized overnight with probes of interest for dual-color FISH, depending on the chromosomes involved (Table 1). The scoring of the fluorescent signals was performed by two independent investigators, using an epifluorescence microscope (Nikon Eclipse 80i or Leica DM 5000B) with adapted filter DAPI, FITC, Orange or triple-band. Manual spot count was performed following strict criteria [7]. Automated FISH results were obtained with Metafer Slide Scanning System and MetaCyte software (Metasystems®, Germany), as reported previously [8], with over one thousand cells analyzed when preparation allowed it.

**Table 1:** Probes used in FISH analysis

Patient	Translocation	Probes
P1 to P9	der(13;14)(q10;q10)	LSI® 13q14 SG (Vysis®, ABBOTT) & TelVysion 14q SO (Vysis®, ABBOTT)
P10 to P14	der(13;15)(q10;q10)	LSI® 13q14 SG (Vysis®, ABBOTT) & TelVysion 15q SO (Vysis®, ABBOTT)
^a^P10 and P11	der(13;15)(q10;q10)	13q32.1 orange (BlueGnome) & CEP 15 SA (Vysis®, ABBOTT)
P15	der(13;22)(q10;q10)	13q32.1 orange (BlueGnome) & LSI® 22 (BCR) SG (Vysis®, ABBOTT)
P16 and P17	der(14;15)(q10;q10)	TelVysion 14q SO (Vysis®, ABBOTT) & CEP 15 SA (Vysis®, ABBOTT)
^a^P16	der(14;15)(q10;q10)	Subtelomere 14q green (Cytocell Aquarius) & CEP 15 SA (Vysis®, ABBOTT)
P18 and P19	der(14;15)(q10;q10)	TelVysion 14q SO (Vysis®, ABBOTT) & CEP 15 SA (Vysis®, ABBOTT)
P20 to P22	der(14;21)(q10;q10)	TelVysion 14q SO (Vysis®, ABBOTT) & Subtelomere 21q green (Cytocell Aquarius)
P23	der(14;22)(q10;q10)	Subtelomere 14q green (Cytocell Aquarius) & Tel22q SO (Amplitech)

^a^patients for whom a second analysis was performed because of insufficient initial count

### Literature analysis

Literature analysis was mainly performed on PUBMED. Searching was performed using the following MESH terms: Robertsonian translocation/sperm FISH/meiotic segregation. A total of 171 publications were found. Among them, 44 publications [9–52] about meiotic segregation of sperm from Robertsonian translocation carriers were found between 1983 and 2017 (Table 2).

**Table 2:** Robertsonian translocation carriers with meiotic segregation analysis in literature

	13;14	14;21	13;15	14;15	14;22	13;21	13;22	21;22	15;22	15;21
*Wang* et al. *2017*	6	3		1			1	1		
*Song* et al. *2016*				1						
*Godo* et al. *2015*	10						1			
*Sobotka* et al. *2015*					1					
*Xu* et al. *2014*				1						
*Perrin* et al. *2013*	1	1	1							
*Pylyp* et al. *2013*	5	3				1				
*Rouen* et al. *2013*	1	1								
*Rouen* et al. *2013*	7	1	1							
*Vozdova* et al. *2013*	11	1							1	
*Bernicot* et al *2012*						1			1	
*Cassuto* et al. *2011*	1	1								
*Ferfouri* et al. *2011*	16	8	1		3					1
*Mahjoub* et al. *2011*	5									
*Anton* et al. *2010*		3					1			
*Brugnon* et al. *2010*	1	1	1		2					
*Perrin* et al. *2010*	3									
*Perrin* et al. *2009*	3	1								
*Nishikawa* et al. *2008*	1	2				1				
*Chen* et al. *2007*	4				1	1				
*Kekesi* et al *2007*	1									
*Brugnon* et al. *2006*	3		1							
*Hatakeyama* et al *2006*						1				
*Moradkhani* et al *2006*			2	2						
*Moradkhani* et al *2006*					3					
*Ogur* et al *2006*	7	2	2	2				1		
*Tang* et al *2006*		1								
*Anahory 2005*							1			
*Rives* et al *2005*			1							
*Roux* et al *2005*	3									
*Anton* et al *2004*	7									
*Frydman* et al *2001*	3	3								
*Morel* et al *2001*	3									
*Escudero* et al *2000*	2									
*Honda* et al *2000*		1								
^*a*^*Ogawa* et al. *2000*	1									
*Mennicke* et al. *1997*								1		
*Rousseaux* et al *1995*		1								
^*a*^*Martin* et al *1992*									1	
^*a*^*Syme* et al *1992*								1		
^*a*^*Pellestor* et al. *1990*			1							
^*a*^*Martin* et al. *1988*	1									
^*a*^*Pellestor* et al. *1987*	1									
^*a*^*Balkan* et al. *1983*		1								
Literature	107	35	11	7	10	5	4	4	3	1
Our study	9	3	5	4	1	0	1	0	0	0
Total	116	38	16	11	11	5	5	4	3	1
*% total*	*55,24*	*18,10*	*7,62*	*5,24*	*5,24*	*2,38*	*2,38*	*1,90*	*1,43*	*0,48*

^a^ *Studies using sperm karyotyping after heterospecific fertilization*

### Data analyzed

Variables analyzed were: sperm concentration (10^6^/ml), motility (%), morphology (%) and meiotic segregation rates of different variants (%).

### Statistical analysis

Data were treated with R software (version number 2.14.1). A probability value of less than 0.05 was considered to be statistically significant.

## Results

### Semen parameters

As summarized in Tables 3, 4 patients were normozoospermic, 6 were oligoasthenozoospermic, 6 were oligoasthenoteratozoospermic (OAT), 3 were oligoteratozoospermic, 2 were asthenozoospermic, and 2 were oligozoospermic.

**Table 3:** Robertsonian translocation carriers age, karyotype and semen parameters

Patient	Age	Karyotype	Semen parameters			Seminogram
			Concentration (×10^6/ml)	Motility (%)	(%) Normal morphology	
P1	36	45,XY,der(13;14)(q10;q10)	4,5	20	4	Oligoasthenozoospermia
P2	26	45,XY,der(13;14)(q10;q10)	0,6	36	3	Oligoasthenoteratozoospermia
P3	34	45,XY,der(13;14)(q10;q10)	0,48	17	3	Oligoasthenoteratozoospermia
P4	38	45,XY,der(13;14)(q10;q10)	28	40	9	Normozoospermia
P5	29	45,XY,der(13;14)(q10;q10)	22,3	50	16	Normozoospermia
P6	33	45,XY,der(13;14)(q10;q10)	2,2	20	2	Oligoasthenoteratozoospermia
P7	38	45,XY,der(13;14)(q10;q10)	25	5	2	Oligoasthenoteratozoospermia
P8	36	45,XY,der(13;14)(q10;q10)	2	30	4	Oligoasthenozoospermia
P9	32	45,XY,der(13;14)(q10;q10)	0.007	17	0	Oligoasthenoteratozoospermia
P10	32	45,XY,der(13;15)(q10;q10)	13	34	8	Oligoasthenozoospermia
P11	27	45,XY,der(13;15)(q10;q10)	23	60	4	Normozoospermia
P12	28	45,XY,der(13;15)(q10;q10)	0,9	48	13	Oligozoospermia
P13	35	45,XY,der(13;15)(q10;q10)	0,02	13	2	Oligoasthenoteratozoospermia
P14	27	45,XY,der(13;15)(q10;q10)	5	45	0	Oligoteratozoospermia
P15	35	45,XY,der(13;22)(q10;q10)	7,3	45	3	Oligoteratozoospermia
P16	38	45,XY,der(14;15)(q10;q10)	27	30	39	Asthenozoospermia
P17	33	45,XY,der(14;15)(q10;q10)	35	30	21	Asthenozoospermia
P18	31	45,XY,der(14;15)(q10;q10)	2,2	30	59	Oligoasthenozoospermia
P19	30	45,XY,der(14;15)(q10;q10)	5	35	15	Oligoasthenozoospermia
P20	40	45,XY,der(14;21)(q10;q10)	42	45	17	Normozoospermia
P21	36	45,XY,der(14;21)(q10;q10)	2,7	40	11	Oligozoospermia
P22	27	45,XY,der(14;21)(q10;q10)	0,4	16	5	Oligoasthenozoospermia
P23	39	45,XY,der(14;22)(q10;q10)	8	45	2	Oligoteratozoospermia

**Table 4:** Meiotic segregation of Robertsonian translocation carriers

Patient	% Alt	% Adjacent				% 3:0	% unbalanced
der(13;14)							
	balanced	disomy 13	nullisomy 13	disomy 14	nullisomy 14	3:0	unbalanced
P1	72.76	6.34	8.21	2.99	6.72	2.99	27.24
P2	71.69	4.57	6.39	7.31	10.05	0.00	28.31
P3	64.94	4.60	15.52	8.62	5.75	0.57	35.06
P4	84.86	2.83	4.66	3.16	4.49	0.00	15.14
P5	85.95	2.42	5.82	2.75	3.07	0.00	14.05
P6	75.39	6.84	6.29	5.74	5.52	0.22	24.61
P7	78.52	7.21	6.38	3.52	4.36	0.00	21.48
P8	66.08	4.59	3.81	9.97	12.12	3.42	33.92
P9	60.69	13.08	3.15	7.75	7.27	8.06	39.31
*Mean*	*73.43*	*5.83*	*6.69*	*5.76*	*6.59*	*1.70*	*26.57*
der(13;15)							
	balanced	disomy 13	nullisomy 13	disomy 15	nullisomy 15	3:0	unbalanced
P10	66.80	6.40	7.60	8.80	10.40	0.00	33.20
P11	68.94	4.61	7.01	4.81	14.63	0.00	31.06
P12	81.32	1.10	6.59	3.30	6.59	1.10	18.68
P13	50.92	9.17	12.84	10.09	16.97	0.00	49.08
P14	81.57	3.66	2.66	3.97	3.76	4.39	18.43
*Mean ± SD*	*69.91*	*4.99*	*7.34*	*6.19*	*10.47*	*1.10*	*30.09*
der(13;22)							
	balanced	disomy 13	nullisomy 13	disomy 22	nullisomy 22	3:0	unbalanced
P15	72.78	4.63	9.51	4.39	8.05	0.73	27.32
der(14;15)							
	balanced	disomy 14	nullisomy 14	disomy 15	nullisomy 15	3:0	unbalanced
P16	71.80	7.00	10.60	3.60	7.00	0.00	28.20
P17	83.90	0.85	4.24	0.21	10.81	0.00	16.10
P18	83.26	2.48	4.75	1.24	8.26	0.00	16.74
P19	68.26	5.49	4.51	7.33	12.56	1.85	31.74
*Mean ± SD*	*76.80*	*3.95*	*6.03*	*3.10*	*9.66*	*0.46*	*23.20*
der(14;21)							
	balanced	disomy 14	nullisomy 14	disomy 21	nullisomy 21	3:0	unbalanced
P20	89.99	3.24	0.99	1.97	3.81	0.00	10.01
P21	53.34	11.35	28.46	3.11	0.00	3.73	46.66
P22	79.71	4.92	7.63	3.73	3.22	0.79	20.29
*Mean ± SD*	*74.35*	*6.51*	*12.36*	*2.94*	*2.34*	*1.51*	*25.65*
der(14;22)							
	balanced	disomy 14	nullisomy 14	disomy 22	nullisomy 22	3:0	unbalanced
P23	76.09	5.07	5.92	4.21	6.78	1.93	23.91

### Sperm FISH analysis

**Fig. 2:**
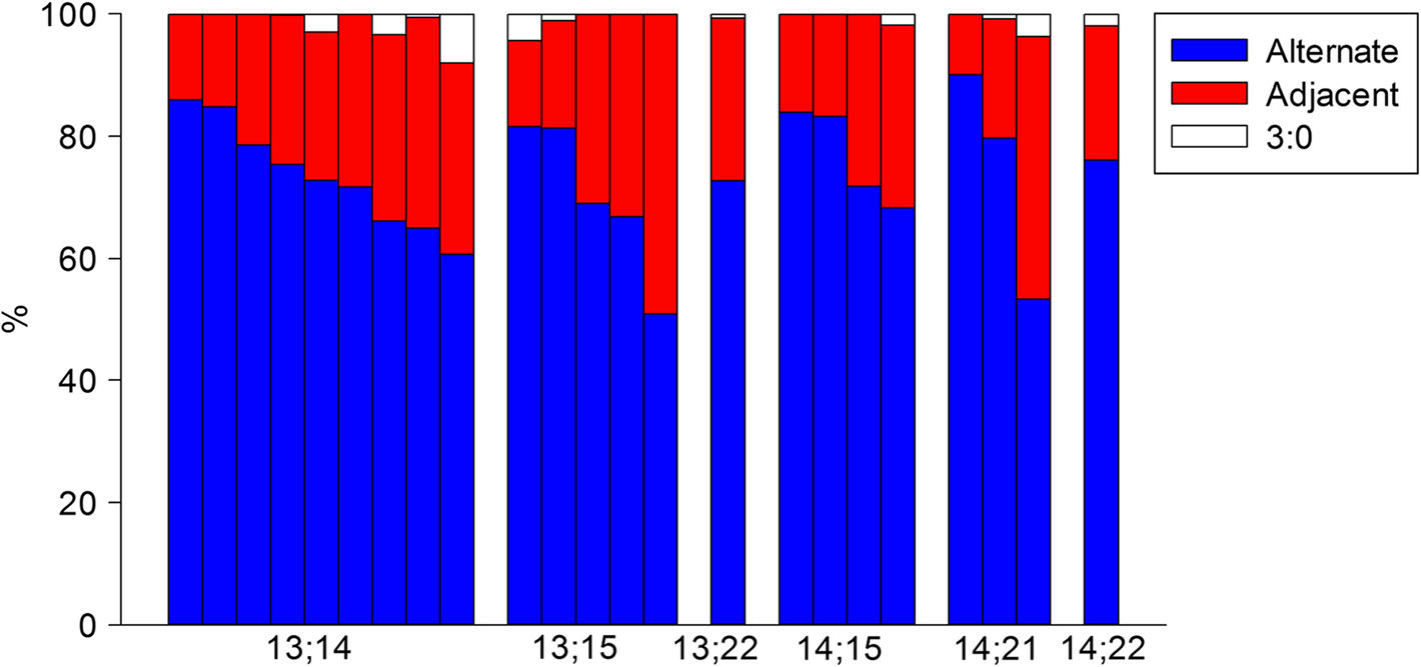
Rates of different variants in meiotic segregation

The number of analyzed sperm ranged from 91 to 1950 for each patient, with a total of 18,261 spermatozoa. Segregation results are illustrated in Fig. 2 and detailed in Table 4 with insight in each mode: alternate, adjacent and 3:0. Our results confirmed a majority of balanced spermatozoa for all patients with a mean ± SE of 73.45 ± 8.05% for all RobT (min 50.92; max 89.99). The rate of unbalanced spermatozoa resulting from adjacent mode of segregation represented 25.25 ± 7.63% (min 10.01%; max 49.08%). The 3:0 segregation mode represented 1.29 ± 1.50% (min 0%; max 8.06%). Mean disomy rates vary from 2.94% to 6.51% (min = 0.21%, max = 13.85%) when comparing all the translocations, while mean nullosomy rates vary from 2.34% to 12.36% (min = 0.00%; max = 16.97%). For each chromosome, mean disomy rate is always lower than mean nullosomy rate.

### Analysis of the segregation data available in the literature

Bibliographic references about sperm FISH analysis of Robertsonian translocation carriers are presented in Table 2. Forty four articles have been published from 1983 to 2017 dealing with meiotic segregation in sperm with thirty nine for the same RobT as in our study. It overall summarized the FISH analysis of 210 patients.

Our segregation rates were compliant with the data from literature for all translocation types (our study versus literature): der(13;14) 73.43 ± 7% versus 83.29 ± 8.72%, der(13;15) 69.91 ± 12.62% versus 79.73 ± 6.73%, der(14;15) 76.80 ± 7.96% versus 84.51 ± 5.58%, der(14;21) 74.35 ± 18.9% versus 83.45 ± 8.3% (*p* > 0.05, t-test). Statistics were not available for der(13;22) and (14;22) as we only added one patient.

**Fig. 3:**
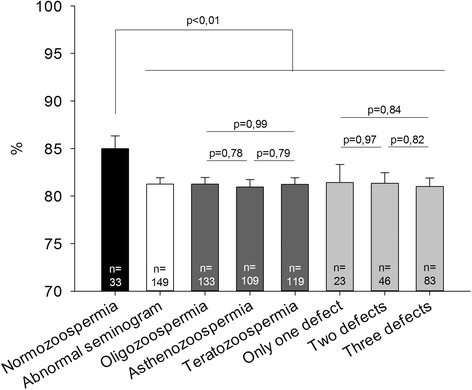
Comparative analysis of the rates of balanced spermatozoa between each RobT. Legend: *n* = number of patients, *p-value < 0.05 versus der(13;15). Statistical analysis not possible for der(15;21) due to the number of patients

Altogether, balanced segregation rates were consistent among the different types of RobT (*p* > 0.05, t-test) except for der(13;15) that exhibited lower balanced spermatozoa rates (Fig. 3). Der(13;15) segregation rates were statistically different (*p* < 0.05, t-test) from those from the two most common Robertsonian translocation der(13;14) and der(14;21), and two less common der(13;21) and der(15;22).

### Correlation between segregation data and semen analysis

From the 187 selected carriers of literature, sperm analysis data were available for 159, and added to our 23 patients. Among all, 33 were normozoospermic (18.13%) and 149 exhibited abnormal seminogram (81.87%). Oligozoospermia was found in 133 patients (73.08%), asthenozoospermia in 109 (59.89%) and teratozoospermia in 119 (65.38%). Twenty three patients had a single anomaly (12.64%), 46 two anomalies (25.27) and 83 displayed OAT (45.60%).

**Fig. 4:**
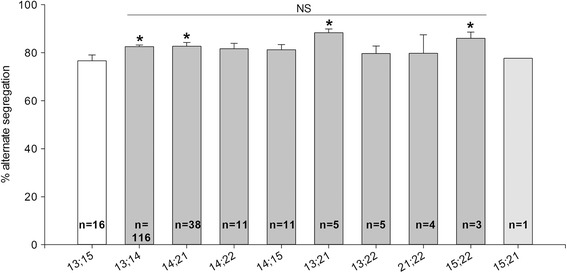
Balanced spermatozoa rates among normozoospermic patients and patients with abnormal seminogram. Legend: n = number of patients

As shown in Fig. 4, normozoospermic patients display a significantly (*p* < 0.01, t-test) higher rate of balanced sperm cells (85%) than patients with seminogram anomalies (81.3%), whatever the number or the type of anomalies involved (*p* > 0.05, t-test).

## Discussion

Thanks to the twenty-three new patients of this study, literature reaches more than two hundred descriptions of meiotic segregation of RobT carriers. Compiling of these data is especially important for rare RobT, like der(13;15) for which we add 5 carriers to the 11 already known (+ 45%), der(14;15) with 4 new patients to the 7 previously published (+ 57%) and der(13;22) with one addition to the 4 patients already presented (+ 25%).

Our rates of balanced segregation (73.45 ± 8.05%) are compliant with the previous studies (*p* > 0.05) for each translocation versus data from publications listed in Table 4, showing the predominance of alternate segregation for all carriers. Similar to literature (41.7%), most of our patients (39.1%) exhibit balanced segregation rates between 75 and 85%. Patients with rates under 65% or over 90% only represent a small proportion of global population both in our study (17.4%) and in literature (16.6%).

It is commonly assumed that rearranged chromosomes of RobT carriers have similar meiotic behavior, regardless of the chromosomes involved ([34], data from 41 carriers). This hypothesis is strongly supported by the similarity of balanced gamete rates among the different RobT carriers. We demonstrate that all RobT segregation rates are similar to each other (*p* > 0.05), except for der(13;15) whose rates are significantly lower (*p* < 0.05) than der(13;14) and der(14;21), the two most frequent translocations, and der(13;21) and der(15;22). The limited size of the cohorts of the other translocations probably explains the lack of significance in segregation rates (*p* = 0.13 vs der(14;15) *n* = 11; *p* = 0.17 vs der(14;22) *n* = 11; *p* = 0.46 vs der(13;22) *n* = 5). No clue has been found so far to explain the difference between der(13;15) segregation rate and the other RobT. It could be due either to the structure of the chromosomes involved in the translocation, or to the spermatogenesis itself. We also clearly show that mean disomy rates are lower than mean nullosomy rates, whatever the chromosome analyzed and that the discrepancies observed among the rates for patients carrying the same translocation are important. When giving genetical counselling to the patients and thinking about preimplantation diagnosis, oocyte fertilization by a nullosomic sperm leads to miscarriage, while oocyte fertilization by a disomic sperm can lead to the birth of a child with MCA-ID. The choice of preimplantation diagnosis may thus be all the more considered as the risk of MCA-ID child is high.

What about spermatogenesis for these patients and the possible links between germ cell production and meiotic segregation? Abnormal semen parameters were found in 82.6% (19 for 23) of our RobT carriers which support the fact that semen parameters of RobT carriers are significantly lower than those of men with normal karyotype [53]. Altered semen parameters have previously been correlated with aneuploidy in RobT carriers [18] and suggested implication in malsegregation rates [21]. Here we confirm that normozoospermic men have higher rates of balanced spermatozoa than men with semen anomalies, whatever the anomaly implied. The proportion of RobT carriers with abnormal seminogram was not different among the translocations analyzed in our cohort (unpublished data) and particularly not between der(13;14) and der(13;15) (*p* = 0.58, Fischer’s exact test). The discrepancies between der(13;15) and the other translocations cannot be explained this way.

It seems also interesting to question if the sperm preparation methods used in assisted reproductive techniques can improve the rates of balanced sperm used in these techniques. Several procedures have been developed to improve the detection or exclusion of sperm with quantitative or qualitative nuclear anomalies (translocation or DNA fragmentation) with partial results [54]. Among them, no morphological discrimination was sufficiently accurate to identify chromosomal imbalances or DNA defects [55, 56], but the use of a simple discontinuous gradient centrifugation could lead to a 30% decrease of unbalanced sperm in chromosomal structural rearrangement carriers [16]. Recent work by Rouen et al [57] suggests that the hypo-osmotic swelling test (HOST) could allow a more efficient selection of balanced sperm in translocation carriers. HOST has already shown some efficacy in normal sperm selection in patients with testicular biopsy and very low sperm count and/or little or no motility, but the efficiency of this procedure has yet to be confirmed under ICSI standard conditions.

Beyond basic cytogenetic research, these data are useful to bring better reproductive and genetic counseling when couples are engaged in PGD. Studies involving PGD for RobT carriers confirmed alternate segregation predominance [58–61]. We consider that sperm FISH is a useful tool to help the management of PGD attempts.

## Conclusion

According to the discrepancies observed between der(13;15) and all the other Rob T carriers, the differences observed among patients presenting normal and abnormal sperm parameters and the input in genetical counselling, sperm FISH does not seem obsolete for these patients. Moreover, it seems important to collect more data for rare RobT.

## Abbreviations

FISH: Fluorescence In-Situ Hybridization
HOST: Hypo-Osmotic Swelling Test
MCA-ID: Multiple Congenital Anomalies and Intellectual Disability
OAT: OligoAsthenoTeratozoospermia
PGD: Preimplantation Genetic Diagnosis
RobT: Robertsonian translocation
SSC: Saline-Sodium-Citrate buffer
WHO: World Health Organization
